# Mechanical and Environmental Properties of Cemented Paste Backfill Prepared with Bayer Red Mud as an Alkali-Activator Substitute

**DOI:** 10.3390/ma18204712

**Published:** 2025-10-14

**Authors:** Lihui Gao, Haicheng Zhao, Nan Guo, Xinmeng Jiang, Yijing Zhang

**Affiliations:** 1 State Key Laboratory of Water Resource Protection and Utilization in Coal Mining, Beijing 102211, China; 2 School of Environment Science and Spatial Informatics, China University of Mining and Technology, Xuzhou 221116, China; 3 National Institute of Clean-and-Low-Carbon Energy (NICE), Beijing 102211, China

**Keywords:** red mud, backfilling material, hydration, compressive strengths, heavy metals, immobilization

## Abstract

This study developed a sustainable high-strength coal gangue backfill material for underground mining applications using coal gangue, fly ash, and cement as primary raw materials, with red mud (RM) as an alternative alkali activator. The mechanical properties of the backfill material were systematically optimized by adjusting coal gangue particle size and alkali activator dosage. The optimized formulation (coal gangue/fly ash/cement = 5:4:1, 3–6 mm coal gangue particle size, 5% RM, which named BF-6-5RM) achieved superior compressive strengths of 8.23 MPa (7 days) and 10.5 MPa (28 days), significantly exceeding conventional backfill requirements and outperforming a CaO-activated reference system (coal gangue/fly ash/cement = 5:4:1, 3–6 mm coal gangue particle size, 2% CaO, which named BF-6-2CaO). Microstructural and physicochemical analyses revealed that both formulations produced calcium silicate hydrate gels (C-S-H gels) and ettringite (AFt) as key hydration products, though BF-6-5RM exhibited a denser microstructure with well-developed ettringite networks and no detectable portlandite (CH), explaining its enhanced early-age strength. Environmental assessments confirmed effective heavy metal immobilization via encapsulation, adsorption, precipitation and substitution, except for arsenic (As), which exceeded Class III groundwater thresholds (DZ/T 0290-2015) due to elevated raw material content, displaying “surface wash-off, diffusion and depletion” leaching behavior. The findings confirm that red mud-based alkali activation is a viable technology for underground backfilling, provided it is coupled with arsenic control strategies like chemical stabilization or the selection of low-arsenic raw materials. This approach not only enables the resource utilization of hazardous industrial waste but also facilitates the production of backfill materials that combine both mechanical strength and environmental compatibility, thereby delivering dual economic and ecological benefits for sustainable mining practices.

## 1. Introduction

The exploitation of coal resources generates substantial amounts of solid waste, including coal gangue and fly ash. Currently, China’s accumulated stockpile of coal gangue has exceeded 6 billion tons, making it the largest industrial solid waste in the country, with annual emissions increasing by approximately 500–800 million tons per year [1]. Meanwhile, fly ash, a byproduct of coal combustion, accounts for 60–88% of the total solid waste generated by coal-fired power plants [2]. For instance, the coal mining, processing and burning generated 795 million tons of coal gangue and 650 million tons of fly ash, presenting significant challenges for waste management and utilization [3]. Backfill mining technology, which involves emplacing engineered materials into excavated voids following coal extraction, represents a proven approach to enhance mineral resource recovery efficiency [4]. The cemented backfill material primarily utilizes coal gangue as aggregate, mixing it with fly ash, cement and water to form a paste-like slurry, and then utilizing pipelines to pump or gravity flow to backfill excavated stopes. The implementation of this backfill technology plays an important role in strata stability and mining safety. Beyond mitigating surface environmental impacts through coal gangue dump elimination, it provides effective goaf rehabilitation while substantially diminishing potential surface subsidence hazards [5,6]. However, coal-based solid wastes exhibit inherent limitations in backfill applications, particularly regarding mechanical performance [7,8] and environmental compatibility. These raw materials typically contain elevated heavy metal concentrations and demonstrate inadequate mechanical strength. Furthermore, the cemented backfill undergoes initial leaching by acidic mine water followed by prolonged immersion. This aqueous exposure facilitates the dissolution and migration of heavy metals from the cementitious matrix, posing significant risks of groundwater contamination over time [1,9,10]. Consequently, developing effective strategies to simultaneously enhance the mechanical strength of coal waste-based backfills and immobilize heavy metal ions becomes critically important.

Alkaline additives (e.g., CaO, MgO, or other commercial alkaline agents, like NaOH, Na_2_CO_3_) typically enhance system strength by promoting hydration reactions that form cementitious phases such as calcium silicate hydrate (C-S-H), while simultaneously immobilizing heavy metal ions. Zheng et al. demonstrated that utilizing NaOH and gypsum as combined alkali activators for slag-based cemented paste backfill achieved optimal performance at dosages of 6 wt% NaOH and 20 wt% gypsum, yielding unconfined compressive strength (UCS) values of 2.1 MPa (3 days), 6.2 MPa (28 days), and 7.3 MPa (180 days) [11]. Wang et al. demonstrated that incorporating 30 wt% CaO as an alkaline additive significantly enhanced the mechanical and environmental performance of high-sulfur tailings. The 28-day UCS increased from 0.48 MPa (control) to 1.82 MPa, representing 280% improvement. Concurrently, CaO achieved exceptional immobilization efficiencies for heavy metals: 98.6% for Zn^2+^, 93.2% for Pb^2+^, 89.1% for Cu^2+^, and 85.4% for Fe^3+^, attributed to synergistic mechanisms involving hydroxide precipitation and substitution within C-S-H phases [8]. Therefore, the incorporation of alkaline additives not only enhances UCS by improving the hydration environment and accelerating reaction kinetics but also facilitates heavy metal immobilization through ion exchange and hydroxide complex precipitation. This dual functionality highlights the significant potential of alkaline additives in optimizing the performance of gangue-based backfill materials.

Red mud (RM), a highly alkaline byproduct (pH 10.0–12.5) of bauxite processing via the Bayer method, is generated at a rate of 0.7–2.0 tons per ton of alumina produced [12]. This yield ratio translates to an estimated global RM output of 120 million tons annually, creating significant challenges for sustainable waste management [13]. Due to its inherent alkaline composition, which is comparable to commercial activators such as sodium hydroxide and sodium silicate, red mud has emerged as an important alternative raw material for producing alkali-activated materials. Recent studies by Li et al. [12] and Liu et al. [14] have demonstrated that partially replacing traditional activators with red mud not only reduces material costs but also enhances the mechanical properties and long-term durability of backfill materials, thereby contributing to improved compressive strength and extended service life in mining applications. However, despite these promising findings, red mud contains higher levels of heavy metals and impurities along with lower alkali purity compared to conventional activators, resulting in fundamental differences in reaction mechanisms. Key aspects such as reaction kinetics and gel phase evolution remain systematically unresolved. Future research should focus on elucidating these distinct reaction pathways to optimize the formulation of red mud-based alkali-activated materials and facilitate their large-scale industrial adoption.

This study investigates the potential of using RM/CaO as an alkaline activator for coal gangue-fly ash-based composites in mining backfill applications. By comparing the UCS value and heavy metal immobilization efficiency of different formulations, the influence of various alkaline activators on the hydration process of cementitious materials was elucidated. Furthermore, the activation mechanisms of the RM/CaO system were revealed through physicochemical characterization of the cementation product formation process. The findings not only clarify the resource utilization pathway for solid wastes in cemented backfilling, but also provide both theoretical foundation and technical support for green transformation in mining engineering and ecological environmental protection.

## 2. Materials and Methods

### 2.1. Raw Materials

Coal gangue, fly ash, cement, RM and CaO were the main raw materials used in the study. The coal gangue and fly ash samples used in this study were sourced from the Shendong mining area, Ordos, Inner Mongolia, China. RM was obtained from an aluminum oxide plant in Zhengzhou, Henan, China. Their chemical composition was analyzed using X-ray fluorescence (XRF), as shown in Table 1. RM primarily consists of Fe_2_O_3_, Al_2_O_3_, SiO_2_, CaO, and Na_2_O, accounting for over 95% of the total composition. The chemical compositions of coal gangue and fly ash are essentially similar, primarily consisting of approximately 50% SiO_2_, 27% Al_2_O_3_, and 10% Fe_2_O_3_.

The particle size distribution of CaO, RM, cement and fly ash was evaluated using a laser particle size analyzer, with a measurement range of 0.1–1200.0 μm, and the results are shown in Table 2. It can be seen from Table 2 that the particle size relationship among them is: Fly ash > CaO > RM > Cement. Meanwhile, according to literature studies [12,13,15,16], the specific surface areas of these materials exhibit the relationship: RM (4000 m^2^/kg) > Fly ash (480 m^2^/kg) > Cement (340 m^2^/kg) > CaO (15 m^2^/kg).

### 2.2. Sample Preparation

The coal gangue was first crushed and sieved into different particle size fractions (Photographs of the coal gangue fractions with different particle sizes can be found in Figure S1): <1 mm, 1–3 mm, 3–6 mm, and >6 mm. The solid matrix of the backfill material was formulated based on an optimal mass ratio reported in the literature [1], consisting of 50% coal gangue, 40% fly ash, and 10% cement. For the chemical activators, the dosages of CaO (1–5%) and RM (2–10%) were selected with the aim of maximizing solid waste utilization and evaluating the feasibility of replacing CaO with RM, drawing upon the broad dosage ranges (0–40% for RM, 0–5% for CaO) found in prior studies [1,12,17]. The mixtures were prepared by adding water to achieve a slurry with a solid content of 75% by mass. This slurry was then cast into 70.7 mm cubic molds for subsequent tests [18]. After 24 h of curing, the specimens were demolded and placed in a controlled chamber maintained at (20 ± 2) °C with a relative humidity of ≥95% for further curing. The detailed mix proportions are provided in Table 3.

### 2.3. Test Methods

#### 2.3.1. Uniaxial Compressive Strengths (UCS)

The UCS of the mortar specimens was evaluated using a WDW-20 electromechanical testing system (South Star Testing Technology Co., Ltd., Shenzhen, China) with 20 kN capacity at designated curing periods (7 and 28 days). All tests were conducted under displacement control at a constant rate of 0.5 mm/min according to the standard procedure [19]. Triplicate measurements were performed for each mixture to ensure data reliability.

#### 2.3.2. X-Ray Diffraction Analysis (XRD)

The mineral phase characterization of cured specimens was conducted by X-ray diffraction (XRD) analysis using a D8 DaVinci diffractometer (Bruker, Billerica, MA, USA) equipped with Cu Kα radiation (40 kV, 40 mA). Scans were performed over a 2θ range of 10–80° with an incremental step size of 0.02°. The MDI JADE software package (Jade 9) facilitated the identification of hydration product compositions.

#### 2.3.3. Scanning Electron Microscope (SEM)

Microstructural characterization of 28-day cured back-filling samples was performed using scanning electron microscopy (SEM) coupled with energy-dispersive X-ray spectroscopy (EDS). Sample preparation involved gold sputter-coating to enhance surface conductivity prior to imaging. EDS analysis was explored at an accelerating voltage of 20 kV.

#### 2.3.4. FTIR Analysis

To determine the changes in the molecular structure of the material before and after the hydration reaction, FTIR analysis was performed and recorded between 4000 and 400 cm^−1^ on a Nicolet 380 Thermo Scientific spectrometer (Thermo Fisher Scientific, Waltham, MA, USA).

#### 2.3.5. Toxicity Leaching Tests

The standard cured 28-day specimens were subjected to heavy metal leaching tests to evaluate the environmental impact of backfill materials under water immersion conditions. The experiments were conducted in compliance with the Solid waste-Extraction procedure for leaching toxicity-Horizontal vibration method (HJ557-2009) [20]. The leaching extracts were prepared at a solid-to-liquid ratio of 1:5. The mixture was then placed on a horizontal oscillator and agitated for 6 h with an amplitude of 40 mm and an oscillation frequency of 110 ± 10 cycles per minute, followed by a 16 h settling period. The supernatant was collected, filtered through a 0.45-μm membrane, and used as the test solution for heavy metal concentration determination.

#### 2.3.6. Leaching Behavior of Bulk Materials

To simulate the long-term contaminant release characteristics of the backfilling material, the standard cured specimens were immersed in deionized water at a solid-to-liquid ratio of 1:5 in accordance with Chinese National Standard GB/T 7023-2011 [21], ensuring that the leaching agent covered the specimens by at least 1 cm. The leaching test was then conducted, with the leaching solution extracted and replaced on days 0.25, 1, 2.25, 4, 9, 16, 36, and 64 of the experiment [22]. Each time, the specimens were transferred to a new container filled with an equal volume of fresh leaching agent, while the original leachate was collected for analytical testing. An aliquot of leachate was filtered and tested via ICP-OES.

By calculating the cumulative release flux (*ε_m_*) of heavy metals at any given time and the segmented leaching slope (r_c(a~b)_), the leaching release mechanisms of heavy metal ions were analyzed (specific calculation steps are provided in the SI). In this study, r_c(1~4)_, r_c(2~5)_, r_c(3~6)_, r_c(2~7)_, r_c(4~7)_, and r_c(5~8)_ were used to represent the leaching behavior during the initial, middle, and later stages, respectively [1], as shown in Table 4.

## 3. Results and Discussion

### 3.1. Mechanical Property

#### 3.1.1. Effect of Coal Gangue Particle Size

**Figure 1:**
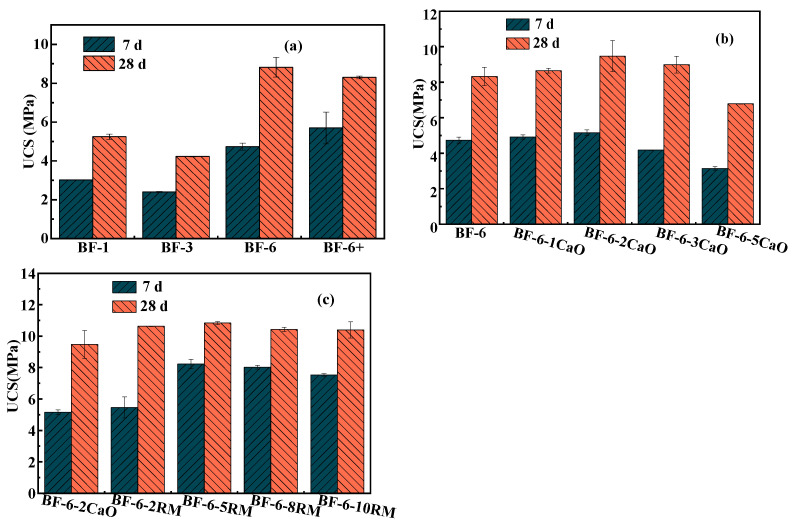
Uniaxial compressive strength of backfill samples of different groups: (**a**) Different particle sizes of coal gangue; (**b**) CaO as activator with different ratios; (**c**) RM as activator with different ratios.

Figure 1a shows the 7 d and 28 d UCS values of coal gangue with different particle sizes mixed with fly ash and cement at a ratio of 5:4:1 without an alkaline activator. We can see the compressive strength of the specimens increases with the extension of curing time, increasing by 70–110% from 7 to 28 days. This suggests that the degree to which compressive strength responds to longer curing periods differs among the particle-size fractions, with the 3–6 mm fraction showing the greatest responsiveness (increased from 4.735 MPa to 8.82 MPa) and the <1 mm fraction the least. Therefore, prolonged curing time is especially beneficial for increasing the compressive strength of backfill whose aggregate spans 3–6 mm. This can be ascribed to their initially high porosity, which depresses early-age strength but also provides ample space for subsequent precipitation of hydration phases; as curing progresses, these products progressively occlude the void network, driving a steeper post-curing strength increase than observed in finer or broader gradings [23,24]. However, for cementitious composites incorporating coal-gangue aggregates finer than 1 mm or in the 1–3 mm range, the compressive strengths at both 7 d and 28 d (≈3 MPa and 5 MPa, respectively) are markedly lower than those containing 3–6 mm or >6 mm fractions. This strength loss is primarily attributed to the excessive specific surface area of the finer particles, which adsorb a disproportionate amount of free water, impair paste workability, and introduce casting defects, ultimately compromising mechanical performance [25,26]. Consequently, coal-gangue aggregate with a particle-size fraction of 3–6 mm was selected as the sole aggregate for subsequent investigations into the effects of different alkaline activators on the cementitious matrix.

#### 3.1.2. Effect of CaO Ratio

As an alkaline additive, CaO plays an important role in improving the hydration environment. Figure 1b illustrates the influence of varying CaO dosages on the cementitious material. The results indicate that as the CaO content increases, the 7-day and 28-day USC values first rise and then decline, reaching their maxima of 5.155 MPa and 9.47 MPa, respectively, at a CaO dosage of 2%. This behavior is mainly attributed to the reaction of CaO with water to produce Ca(OH)_2_, which elevates the alkalinity of the system. Under strong alkaline conditions, the aluminosilicate glass phases in fly ash and coal gangue dissolve, breaking some Si-O and Al-O bonds. The released Si and Al then react with Na^+^ and OH^−^ to form Si- and Al-based oligomers. These oligomers subsequently transform into zeolite-like aluminosilicates and eventually dehydrate to form amorphous gel phases [16]. With further addition of CaO, the compressive strength declines slightly, likely due to excess Ca^2+^ reacting to form Ca(OH)_2_ rather than participating in hydration reactions to produce CSH gel and other hydration products. This prevents the cementitious matrix from restricting the volume expansion of Ca(OH)_2_ crystals, leading to initial microstructural damage and ultimately lower macroscopic strength [27]. Consequently, the mixture containing 2% CaO was selected for comparative studies with red-mud-based alkali-activated binders.

#### 3.1.3. Effect of RM Ratio

Figure 1c presents the compressive strength of geopolymers with varying RM contents as an alkali activator. As shown in the figure, the 7-day and 28-day compressive strengths exhibit distinct trends with increasing RM dosage. At 7 days of curing, the compressive strength initially increases and then decreases with higher RM incorporation, reaching a maximum of 8.23 MPa at 5% RM. This enhancement is attributed to the high Na_2_O and CaO content (8.4% and 18.9%) in RM [28], which releases abundant OH^−^ upon hydration, rapidly elevating the system pH. This alkaline environment disrupts the Si-O-Al networks in precursors (e.g., fly ash and slag), promoting the dissolution of reactive Si^4+^ and Al^3+^ to form oligomers that subsequently condense into C-(A)-S-H or N-A-S-H gels [13]. Additionally, fine RM particles (typically <50 μm) fill the interparticle voids in the geopolymer matrix, reducing porosity, while their hydration products contribute to a denser microstructure, thereby improving mechanical strength. However, excessive RM (>5%) leads to a relative deficiency of reactive aluminosilicate sources and promotes the early formation of coarse crystalline phases (e.g., dicalcium silicate, ettringite) under high alkalinity, ultimately reducing the 7-day strength.

In contrast, at 28 days of curing, the influence of RM content on compressive strength diminishes, with all samples stabilizing at approximately 10.5 MPa. This convergence suggests that early-age strength variations are primarily governed by alkali activation kinetics and the availability of reactive components, whereas prolonged hydration and the micro-aggregate effect homogenize the mechanical performance over time [29].

Given that the compressive strengths at 28 days of curing were comparable (and optimal within the group) when using CaO and RM as activators at 2% and 5% dosages, respectively, the subsequent study selected BF-6-2CaO and BF-6-5RM for characterization of the cementitious materials, as well as investigation of heavy metal leaching behavior and immobilization mechanisms.

### 3.2. Backfilling Material Characteristics

#### 3.2.1. Mineralogical Analysis

**Figure 2:**
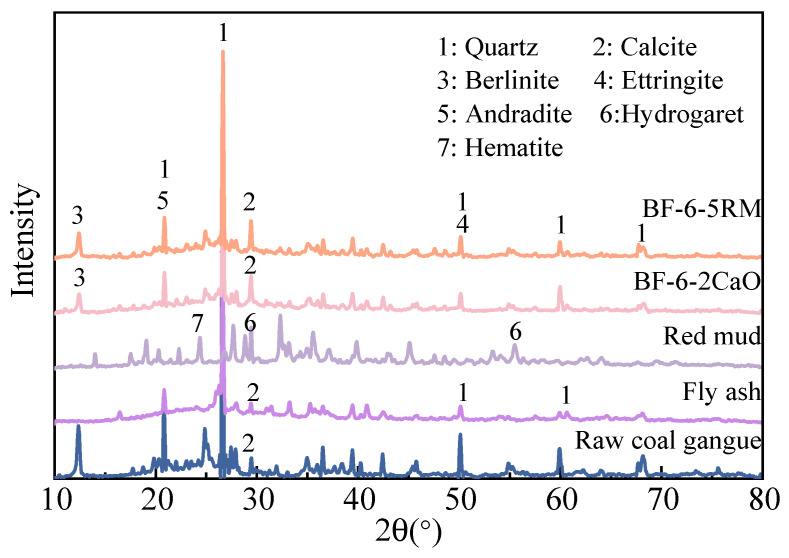
XRD patterns of the raw and backfilling samples at 28 days.

Figure 2 shows the XRD results of raw samples and backfilling samples after 28 days. The XRD curves for BF-6-2CaO and BF-6-5RM present similar patterns. In the hydration products of the two backfilling samples, identified crystalline phases include quartz, calcite and Berlinite. Notably, calcite in two backfilling samples shows sharper diffraction peaks compared to raw coal gangue. This is mainly due to the fact that during the curing process, as the hydration reactions proceed, the calcite in the raw materials (such as gangue and fly ash) undergoes a recrystallization process under the activation of alkali additives, resulting in more complete and regular crystals and thus higher crystallinity of calcite. Although XRD can characterize crystalline products in detail, it has limitations in identifying amorphous products formed during the hydration process (including cement hydration, pozzolanic reaction, and geopolymerization reaction), such as C-S-H gel or C-A-S-H gel [17]. Complementary experimental techniques (SEM and FTIR) are needed to further characterize these amorphous products in order to fully understand their role in the strength development and formation of the cementitious material [15].

#### 3.2.2. Microstructural Analysis

**Figure 3:**
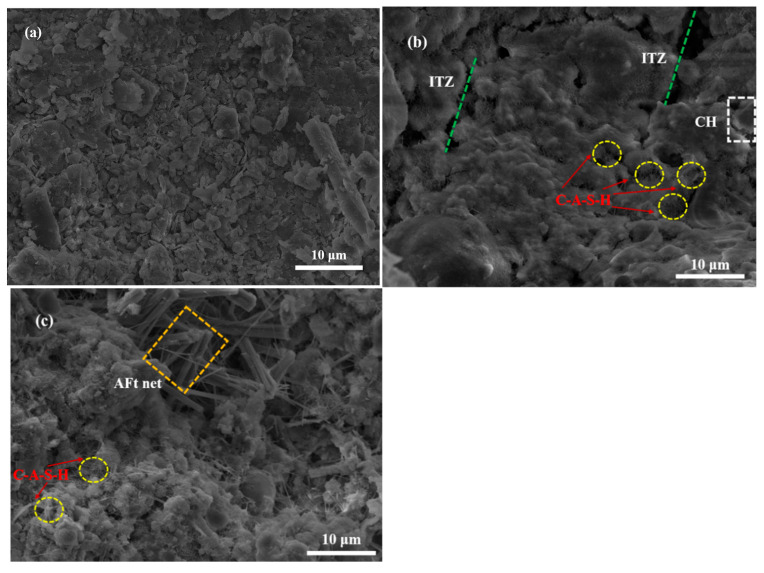
SEM micrographs of coal gangue-based back-filling samples after 28 d of curing (**a**) Raw coal gangue; (**b**) BF-6-2CaO; (**c**) BF-6-5RM.

To further investigate the role of RM as an alternative alkali activator, the microstructure of samples cured for 28 days was analyzed using SEM, as illustrated in Figure 3. The results revealed that raw coal gangue particles were dispersed with a rough surface, whereas the backfilling samples of BF-6-2CaO and BF-6-5RM developed a relatively dense microstructure after curing. Notably, a weak interfacial transition zone (ITZ) between aggregates and the cement paste was observed in the BF-6-2CaO sample (Figure 3b), which typically serves as a preferential path for crack propagation under mechanical loading. In contrast, the BF-6-5RM sample exhibited superior bonding with aggregates, showing no distinct ITZ, thereby providing a microstructural explanation for its measured 28-day compressive strength being 14.4% higher than that of BF-6-2CaO.

Morphologically, the BF-6-2CaO sample was filled with abundant flocculent and fibrous C-S-H or C-A-S-H gels, along with exposed hexagonal plate-like portlandite (CH) crystals. The presence of CH suggests that the sample retained potential for further geopolymerization reactions [30], yet its crystalline morphology contributes less to strength development compared to amorphous gel phases. In the BF-6-5RM sample (Figure 3c), the microstructure appeared more compact, with well-developed cylindrical ettringite (AFt) networks and no exposed CH crystals. This indicates that under the high-alkali environment facilitated by RM, CaO from coal gangue and fly ash dissolved effectively, reacting with liberated SiO_2_ and Al_2_O_3_ to form additional C-S-H or C-A-S-H gels during geopolymerization [31]. These findings align with the mechanical strength development trends observed in the different filler samples. These findings align with the mechanical strength development trends observed in the different filler samples, establishing a direct structure-property relationship where the compact, homogeneous microstructure with eliminated ITZ and well-developed gel networks in BF-6-5RM quantitatively explains its superior mechanical performance.

#### 3.2.3. FTIR Analysis

**Figure 4:**
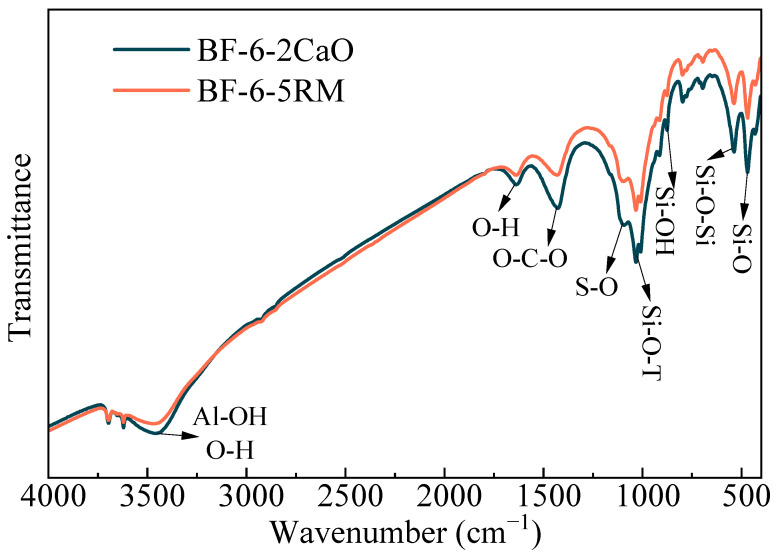
FTIR spectra of backfilling samples BF-6-2Cao and BF-6-5RM.

Figure 4 shows the FTIR spectra of coal gangue-based backfilling samples with different alkali additives. The FTIR spectra of the two samples were highly similar, with virtually identical absorption bands. The band at 3440 cm^−1^ is assigned to the O–H stretching vibration in hydration products and to the Al–OH vibration in ettringite [32,33]. The peak at 1625 cm^−1^ corresponds to the O–H stretching vibration in hydration products, whereas the band at 1465 cm^−1^ arises from the asymmetric stretching of CO_3_^2−^ [34], primarily reflecting carbonation during curing. The absorption at 1114 cm^−1^ is characteristic of SO_4_^2−^ [32], whereas the band at 997 cm^−1^ is associated with the asymmetric stretching of Si–O–T (T = Si, Al) bonds [35], indicating the presence of both C–S–H and C–A–S–H gels in the samples. The peak near 874 cm^−1^ originates from the Si–OH stretching vibration, and the band at 564 cm^−1^ corresponds to the Si–O–Si vibration [15]. Finally, the absorption at 473 cm^−1^ is related to the asymmetric bending vibration of Si–O bonds in the raw materials [36] and is also characteristic of C–S–H. These FTIR results are consistent with the preceding XRD and SEM analyses, further confirming that RM can serve as an effective alkaline activator in the synthesis of cementitious materials.

### 3.3. Analysis of the Economic Environment

#### 3.3.1. Analysis of Heavy Metal Concentrations

Table 5 lists the ion concentrations measured after 6 h leaching (solid-to-liquid ratio 1:10, Deionized water) of raw coal gangue, raw fly ash, BF-6-2CaO and BF-6-5RM. The toxicity-characteristic leaching results show that the contents of As, Pb, Cr, Mn and Ni in the untreated raw materials exceed the limits for Class III groundwater specified in the Chinese standard DZ/T 0290-2015 [37]. Leachates from standard-cured specimens (BF-6-2CaO and BF-6-5RM) exhibit concentrations of all heavy-metal ions below the regulatory thresholds except for As. This can be attributed to the inherent As content in the raw materials, particularly the higher levels found in coal gangue and fly ash. Notably, the formulation activated with RM (BF-6-5RM) exhibited a slightly higher As leaching concentration (0.65 mg/L) than the CaO-activated control (BF-6-2Cao, 0.57 mg/L). This phenomenon resulted from the mobilization of As via three synergistic mechanisms: firstly, the alkaline-oxidative environment during curing destroyed the crystal structure of arsenian pyrite (Fe(As,S)_2_)—the primary host for As in coal gangue—releasing As into the leachate [38,39]. Secondly, the strongly alkaline conditions destabilized As originally encapsulated within aluminosilicate glass phases in the fly ash, facilitating its dissolution [40]. Finally, the same alkaline environment induced the desorption of arsenate (AsO_4_^3−^) and arsenite (AsO_3_^3−^) anions from the iron/aluminum (hydr)oxides in the RM activator, where they were previously bound by surface complexation. The combined effect of these processes significantly enhanced the mobility and leaching of As from the final cementitious matrix.

After 28 d of curing, the leached concentrations of heavy metals from BF-6-2CaO and BF-6-5RM were markedly lower than those from the corresponding raw materials. This reduction is chiefly ascribed to the entrapment of the native heavy metals within the solid matrix of the backfilling specimens by hydration products (such as calcium hydroxide, AFt, and C–S–H gel) [13]. Such encapsulation makes it difficult to migrate or release of these metals, decreasing the possibility of their leaching into the aqueous phase. Meanwhile, with the porous micro-structure and ion-exchange capacity, AFt further contributes by adsorbing or immobilizing heavy-metal ions [41]. In addition, C–S–H gel, the dominant hydration phase, has a negatively charged surface that electrostatically attracts and adsorbs cationic metal species (e.g., Pb^2+^ and Cd^2+^). Through adsorption and ion-exchange mechanisms, C–S–H effectively captures heavy metals and suppresses their migration [42]. The small amounts of Cd, Cu and Zn present in the raw materials can precipitate as insoluble compounds like Cd(OH)_2_, Cu(OH)_2_ and Zn(OH)_2_ under alkaline conditions, whereas Pb reacts with dissolved Si to form Pb_3_O_5_ to further decrease the heavy-metal concentrations in the alkaline environment [43].

#### 3.3.2. The As Leaching Mechanisms of Backfilling Samples

Leachate analysis of raw and backfilling materials after 6 h immersion showed that only As exceeded the Class III groundwater thresholds (DZ/T 0290-2015). Therefore, to examine the As leaching mechanism in BF-6-2CaO and BF-6-5RM, its concentration was monitored in a semi-dynamic immersion test.

**Figure 5:**
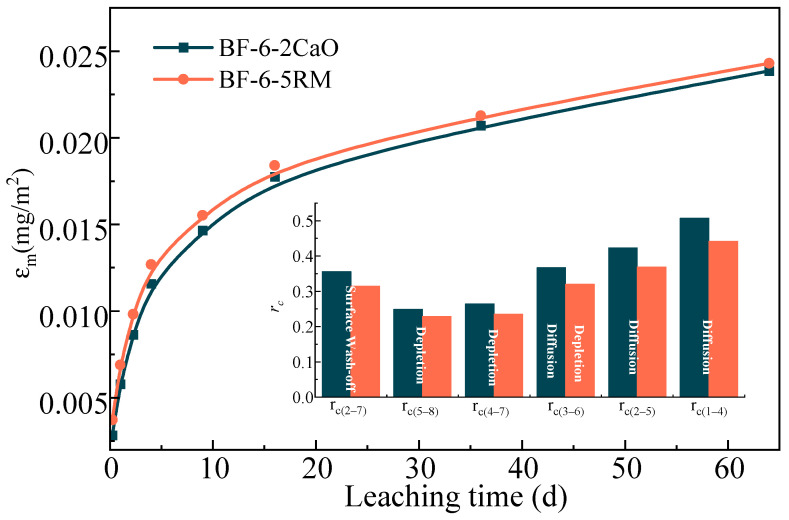
Leaching mechanism of As in backfilling samples BF-6-2Cao and BF-6-5RM.

Figure 5 shows the cumulative release of As from BF-6-2CaO and BF-6-5RM, as a function of leaching time. The overall leaching trend was similar for both materials. The cumulative As release increased rapidly during the initial 9 d, followed by a more gradual increase until approximately 36 d, after which the release rate significantly decreased, approaching a near-equilibrium state. Notably, the cumulative As release from BF-6-5RM was marginally higher than that from BF-6-2CaO throughout the leaching period, suggesting a slight influence of the material composition on the As immobilization capacity.

The inset schematic diagram delineates the internal As leaching mechanisms, which can be interpreted as three consecutive stages [1,22]. Initial Stage (0–9 d): Dominated by Surface Wash-off and Diffusion. The rapid increase in As concentration is primarily attributed to the immediate dissolution and wash-off of weakly bound As from the particle surfaces and macropores. Concurrently, the steep concentration gradient between the solid matrix and the leachant drives the initial fast diffusion of readily available ions from the particle surface and larger pores. Intermediate Stage (9–36 d): Controlled by Diffusion. As the readily accessible surface As is depleted, the release rate slows and becomes governed by the slower process of diffusion. This stage is characterized by the diffusion of As ions from the smaller, less accessible pores within the particle matrix to the solid-leachant interface, which acts as the rate-limiting step. Final Stage (After 36 d): Characterized by Depletion. The system approaches a dynamic equilibrium where the leachable As pool is substantially exhausted. The release rate diminishes to a minimum as the remaining As is either stably incorporated within the solid matrix or is accessible only through extremely slow diffusion pathways, leading to a plateau in the cumulative release curve.

#### 3.3.3. Examination of Heavy Metal Solidification Mechanism

**Figure 6:**
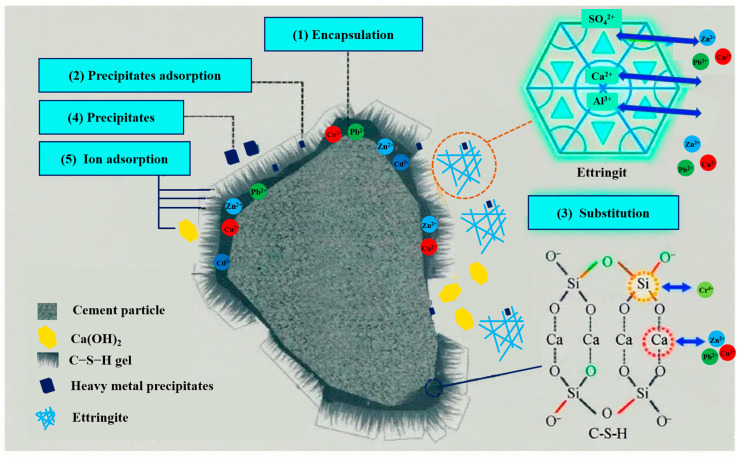
Mechanism of heavy metals stabilized by cementitious materials.

In this study, red mud (RM), coal gangue, fly ash, and cement were used as raw materials. So, the immobilization mechanisms of heavy metals in coal gangue-based cementitious materials can be understood and interpreted based on the typical solidification principles observed in cement-based materials. As shown in Figure 6, the immobilization of heavy metals in cementitious materials is a complex physicochemical process that reduces their mobility through mechanisms including physical encapsulation, chemical reactions, adsorption, and ion exchange [1,13,44,45].

The surfaces of BF-6-2CaO and BF-6-5RM were covered with abundant hydration products, forming a physical encapsulation effect. The primary hydration product, calcium silicate hydrate (C–S–H), is an amorphous or microcrystalline gel with a complex layered structure containing numerous nanoscale pores between its layers, thereby exhibiting significant adsorption capacity [46]. Its abundant surface active sites adsorb heavy metal ions through electrostatic attraction. For instance, heavy metal cations (e.g., Pb^2+^, Cu^2+^, Zn^2+^) can bind to active sites on C–S–H surfaces, such as hydroxyl groups and siloxane bridges, via electrostatic interactions or complexation. Meanwhile, heavy metal anions can first associate with the negatively charged C–S–H surface through Ca^2+^ and subsequently bond with calcium ions [13].

Beyond adsorption, certain heavy metal ions may substitute calcium or silicon within the C–S–H lattice. For example, Pb^2+^, Cu^2+^, and Zn^2+^ can replace Ca^2+^, while Cr^4+^ may substitute Si^4+^ [47]. Although this process is generally slow, long-term immobilization can still be achieved for metals with smaller ionic radii and compatible chemical environments. Additionally, heavy metal ions can react chemically with components in C–S–H, such as OH^−^ or SiO_4_^4−^, forming insoluble precipitates (the leachate pH > 10 from both cementitious materials indicates an alkaline environment conducive to such reactions). This process is particularly effective for heavy metals prone to forming hydroxides or carbonates precipitates (e.g., Pb^2+^, Cu^2+^) [48].

## 4. Application Scenario Assessment and Recommendations

Based on the As concentration in the BF-6-5RM leachate and its leaching mechanism, and considering environmental factors such as pH, redox conditions, and potential water contact in different engineering application scenarios, the risk levels are evaluated as shown in Table 6. Therefore, RM-activated coal gangue-based cementitious materials still pose a potential risk of gradual As release during backfilling operations. It is recommended to adopt chemical stabilization and solidification methods for underground backfilling, such as: (1) Iron/Aluminum Salt Treatment [49,50]: Add iron sulfate (Fe_2_(SO_4_)_3_) or ferric chloride (FeCl_3_) into the mixture. The (hydr)oxides produced by the hydrolysis of iron and aluminum can efficiently adsorb or co-precipitate arsenic, forming stable minerals (e.g., scorodite). (2) Phosphate Treatment: Introduce phosphates such as calcium dihydrogen phosphate, which can compete with arsenic for adsorption sites and promote the formation of less soluble calcium arsenate minerals, ensuring long-term stability [51].

Additionally, raw material selection criteria should be strengthened by establishing chemical thresholds for screening, prioritizing the use of pre-treated materials. For instance, using weathered or pre-washed coal gangue, in which unstable sulfides have mostly been oxidized, can reduce the risk of sudden arsenic release at the source.

## 5. Conclusions

This study developed a sustainable high-strength coal gangue backfill material for underground mining using coal gangue, fly ash, and cement as raw materials, with red mud as an alternative alkali activator. The mechanical properties of the backfill material were enhanced by optimizing coal gangue particle size and alkali activator dosage. Microstructural and physicochemical characterization elucidated the hydration reaction mechanism responsible for the improved compressive strength. Furthermore, heavy metal leaching tests confirmed the environmental safety of the material. The results demonstrate the following.

(1) The optimized backfill material formulation (BF-6-5RM, with coal gangue/fly ash/cement ratio of 5:4:1, 3–6 mm coal gangue particle size, and 5% red mud addition), achieved compressive strengths of 8.23 MPa at 7 days and 10.5 MPa at 28 days. These values significantly exceeded conventional backfill strength requirements and outperformed the CaO-activated backfilling material (BF-6-2CaO, 5.155 MPa at 7 days and 9.47 MPa at 28 days).

(2) Physicochemical characterization revealed that both BF-6-2CaO and BF-6-5RM exhibited similar surface functional groups and crystalline phase compositions, with C-S-H gels and ettringite (AFt) identified as the key hydration products responsible for mechanical strength enhancement. However, the BF-6-5RM sample demonstrated a more compact microstructure, featuring well-developed columnar ettringite networks and no exposed portlandite (CH) crystals.

(3) Environmental analysis revealed that the cementitious materials could effectively immobilize heavy metals through encapsulation, adsorption, precipitation, and substitution. However, due to the elevated As content in raw materials, the leachate As concentration exceeded Class III groundwater thresholds (DZ/T 0290-2015). The leaching behavior exhibited a triphasic pattern: initial surface wash-off and diffusion, intermediate diffusion-dominated phase, and final depletion stage.

(4) Arsenic control measures should be implemented for red mud-activated coal gangue backfill to ensure safe application. These include chemical stabilization methods (e.g., iron-aluminum salts or phosphates) and the selection of optimized raw materials (e.g., pre-treated coal gangue), which are crucial for long-term environmental safety in underground backfilling operations.

In conclusion, the utilization of red mud (RM) as an alkaline activator for underground backfill material preparation not only effectively consumes substantial quantities of this strongly alkaline aluminum industry waste but also achieves dual enhancement of economic benefits and environmental performance.

## Figures and Tables

**Table 1:** Chemical composition of raw material (wt%, ND means not detected).

Composition	Coal Gangue	Fly Ash	RM
Na_2_O	0.90 ± 0.035	0.81 ± 0.009	8.42 ± 0.435
MgO	1.46 ± 0.050	1.35 ± 0.042	1.00 ± 0.035
Al_2_O_3_	26.36 ± 1.135	27.92 ± 1.023	22.84 ± 1.257
SiO_2_	50.76 ± 1.035	49.47 ± 1.375	18.07 ± 1.005
P_2_O_5_	0.19 ± 0.041	0.21 ± 0.002	0.22 ± 0.001
SO_3_	1.06 ± 0.077	0.58 ± 0.005	0.82 ± 0.012
Cl	ND	0.06 ± 0.001	0.08 ± 0.002
K_2_O	2.56 ± 0.85	2.38 ± 0.044	2.31 ± 0.089
CaO	4.43 ± 1.321	5.18 ± 0.735	18.98 ± 1.001
TiO_2_	1.28 ± 0.875	1.30 ± 0.017	5.56 ± 0.783
Cr_2_O_3_	0.03 ± 0.004	0.04 ± 0.003	0.10 ± 0.005
MnO	0.12 ± 0.005	0.11 ± 0.002	0.05 ± 0.001
Fe_2_O_3_	10.48 ± 0.877	9.96 ± 0.835	21.028 ± 1.512
CuO	0.02 ± 0.001	0.04 ± 0.001	ND
ZnO	0.15 ± 0.071	0.23 ± 0.008	ND
Rb_2_O	0.03 ± 0.001	0.03 ± 0.001	ND
SrO	0.11 ± 0.002	0.15 ± 0.014	0.28 ± 0.009
ZrO_2_	ND	ND	0.19 ± 0.002
Nb_2_O_5_	ND	ND	0.03 ± 0.001
BaO	0.11 ± 0.005	0.13 ± 0.011	ND
PbO	0.04 ± 0.001	0.05 ± 0.002	0.06 ± 0.001

**Table 2:** Statistics of particle size distribution of raw materials.

Raw Samples	D_10_ (μm)	D_50_ (μm)	D_90_ (μm)	D_av_ (μm)
CaO	12.076 ± 1.089	21.321 ± 1.211	82.189 ± 3.112	46.780 ± 2.012
RM	0.157 ± 0.012	6.743 ± 0.878	47.243 ± 0.748	31.234 ± 1.142
Fly ash	11.082 ± 1.009	34.687 ± 2.114	111.359 ± 4.743	52.410 ± 2.014
Cement	7.068 ± 0.117	18.941 ± 1.621	45.214 ± 1.324	22.149 ± 0.097

Note: D_10_, D_50_, D_90_, and D_av_ are the particle sizes corresponding to the cumulative passing volume percentages of 10%, 50%, 90%, and the average cumulative passing particle size, respectively.

**Table 3:** Summary of back-filling samples design.

Back Filling Samples	Coal Gangue (g)				Fly Ash (g)	Cement (g)	CaO (g)	RM (g)
	<1 mm	1–3 mm	3–6 mm	>6 mm				
BF-1	900				720	180	0	0
BF-3		900			720	180	0	0
BF-6			900		720	180	0	0
BF-6+				900	720	180	0	0
BF-6-1CaO			882		720	180	18	
BF-6-2CaO			864		720	180	36	
BF-6-3CaO			846		720	180	54	
BF-6-5CaO			810		720	180	90	
BF-6-2RM			864		720	180		36
BF-6-5RM			810		720	180		90
BF-6-8RM			756		720	180		144
BF-6-10RM			720		720	180		180

Note: The mixed backfill material was designated as BF-a-bX, where BF represents backfill sample, a denotes the maximum particle size of coal gangue, b indicates the weight ratio of alkaline activator, and X refers to the type of alkaline activator.

**Table 4:** The r_c_ value of heavy metal release behavior at different stages.

Stage	r_c_ ≤ 0.35	0.35 < r_c_ ≤ 0.65	r_c_ > 0.65
r_c(2–7)_	Surface Wash-off	Diffusion	Dissolution
r_c(5–8)_	Exhaustion	Diffusion	Dissolution
r_c(4–7)_	Exhaustion	Diffusion	Dissolution
r_c(3–6)_	Exhaustion	Diffusion	Dissolution
r_c(2–5)_	Exhaustion	Diffusion	Dissolution
r_c(1–4)_	Surface Wash-off	Diffusion	Delayed Diffusion/Dissolution

**Table 5:** Results of leaching (ND means undetected).

Samples	Leaching Ion (mg/L)							
	As	Cr	Ni	Cu	Zn	Cd	Pb	Mn
RM	0.097 ± 0.003	0.370 ± 0.007	0.162 ± 0.003	0.039 ± 0.001	0.144 ± 0.013	0.015 ± 0.001	0.027 ± 0.003	0.385 ± 0.014
Coal gangue	0.380 ± 0.014	0.039 ± 0.001	0.068 ± 0.001	0.078 ± 0.002	0.102 ± 0.021	0.042 ± 0.002	0.017 ± 0.001	0.147 ± 0.023
Fly ash	0.279 ± 9.01 × 10^−3^	0.037 ± 0.001	0.068 ± 0.001	0.083 ± 0.002	0.067 ± 0.002	0.047 ± 0.002	0.008 ± 2.11 × 10^−4^	0.205 ± 0.031
BF-6-2Cao	0.257 ± 0.011	0.0364 ± 0.001	0.001 ± 1.02 × 10^−4^	0.004 ± 1.77 × 10^−4^	ND	ND	0.005 ± 1.21 × 10^−4^	ND
BF-6-5RM	0.265 ± 0.012	0.0381 ± 0.001	0.004 ± 2.21 × 10^−4^	0.007 ± 2.34 × 10^−4^	ND	ND	0.004 ± 9.44 × 10^−5^	ND
DZ/T02902015limits	0.01	0.05	0.02	1	1	0.01	0.01	0.1

**Table 6:** Application Scenarios and Risk Assessment in RM-Activated Coal Gangue Composites.

Application Scenarios	Risk Level	Basis for Risk Assessment
Road Base/Subbase and Fill Materials	Middle-High	Under prolonged exposure to rainwater leaching (under acidic or neutral conditions) and atmospheric oxidation, arsenopyrite in coal gangue will undergo continuous oxidation. Additionally, As present in fly ash and RM may be leached out, posing a potential risk of soil and groundwater contamination.
Underground Backfill	Low-Middle	The underground environment is relatively isolated, characterized by anoxic conditions and stable humidity, which effectively suppresses the oxidation of arsenopyrite, thereby reducing the risk of As release. However, a potential for long-term, slow release remains if water infiltrates the backfill body.
Sintered Building Materials	Low	During the high-temperature sintering process, arsenic is immobilized within the glass phase, forming a stable chemical fixation that significantly reduces its leaching risk. However, strict control over the sintering temperature and atmosphere is essential to ensure this immobilization effect.
Cement Concrete Aggregate (Non-Structural)	Middle	The high-alkalinity environment (pH > 12) generated by cement hydration can temporarily inhibit the leaching of certain arsenic species. However, it may also lead to the dissolution of fly ash glassy phases and the desorption of arsenic adsorbed on red mud. Long-term carbonation (reaction with CO_2_) will cause a pH drop, potentially triggering the delayed release of arsenic.

## Data Availability

The original contributions presented in this study are included in the article/Supplementary Material. Further inquiries can be directed to the corresponding author.

## Supplementary Materials

The following supporting information can be downloaded at https://www.mdpi.com/article/10.3390/ma18204712/s1, Figure S1: Coal gangue fractions with different particle sizes.
