# Supplementary material for: Mechanical and Environmental Properties of Cemented Paste Backfill Prepared with Bayer Red Mud as an Alkali-Activator Substitute
Source: Materials (Basel). 2025 Oct 14;18(20):4712. doi: 10.3390/ma18204712 (PMC12566605; doi:10.3390/ma18204712)
Supplement: Supplementary file 1 [file materials-18-04712-s001.zip › materials-3882502-supplementary.pdf]

Article

Mechanical and Environmental Properties of Cemented Paste Backfill Prepared with Bayer Red Mud as an Alkali-Activator Substitute

Lihui Gao [1,2], Haicheng Zhao [2], Nan Guo [1,3,*], Xinmeng Jiang [2] and Yijing Zhang [2]

[1] State Key Laboratory of Water Resource Protection and Utilization in Coal Mining, Beijing 102211, China
[2] School of Environment Science and Spatial Informatics, China University of Mining and Technology, Xuzhou 221116, China
[3] National Institute of Clean-and-Low-Carbon Energy (NICE), Beijing 102211, China
* Correspondence: 20089952@ceic.com

Text S1: Leaching Behavior of Bulk Materials

Metal release kinetics were quantified for each sampling interval through the following formula:

$$E_j^* = \frac{V_j C_j}{fA} \quad (1)$$

where C_j is the concentration of heavy metals in the j-th leachate (μg/L); V_j is the volume of leachate at the j-th extraction stage (L); f is the conversion factor (1000 μg/mg); A is the specimen surface area (m^2). In addition, the cumulative leaching for each component was subsequently determined through progressive summation using the following formula:

$$\varepsilon_m = \sum_{j=1}^{m} E_j^* \quad (2)$$

where $\mathcal{E}_m$ is the calculated cumulative leaching of a component for period m comprising fraction j=1-m, in mg/m^2; E_j^* is measured according to the formula (1); m is the number of periods equal to the number of he predetermined leachant renewal frequency (where N=8 in this experimental design).

Based on the research results published by Liu et al. [1], the eight leaching stages were categorized into six characteristic intervals. The leaching mechanisms of heavy metals were subsequently analyzed by calculating the linear regression slopes for each defined interval as shown in Equation (3):

$$r_{c(a\sim b)} = \frac{log\varepsilon_a - log\varepsilon_b}{logt_a - logt_b} \quad (3)$$

where the cumulative heavy metal releases (ε_a and ε_b, mg/m^2) represent the total mass fluxes from the initial stage through intervals a and b (where a and b are integers), respectively, while t_a and t_b denote the termination times (s) of leaching phases a and b.

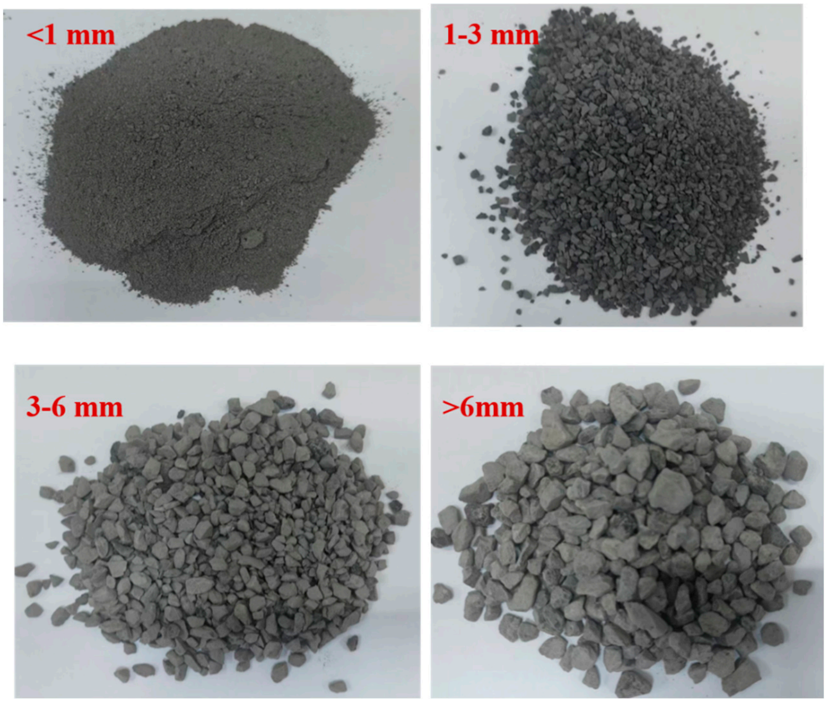

Figure S1. Coal gangue fractions with different particle sizes.
